# Beyond Neurons: Long Distance Communication in Development and Cancer

**DOI:** 10.3389/fcell.2021.739024

**Published:** 2021-09-21

**Authors:** Patrick McMillen, Madeleine J. Oudin, Michael Levin, Samantha L. Payne

**Affiliations:** ^1^Department of Biology, Allen Discovery Center, Tufts University, Medford, MA, United States; ^2^Department of Biomedical Engineering, Tufts University, Medford, MA, United States

**Keywords:** cell signaling, cell network, bioelectricity, tunneling nanotubes, macrophages

## Abstract

Cellular communication is important in all aspects of tissue and organism functioning, from the level of single cells, two discreet populations, and distant tissues of the body. Long distance communication networks integrate individual cells into tissues to maintain a complex organism during development, but when communication between cells goes awry, disease states such as cancer emerge. Herein we discuss the growing body of evidence suggesting that communication methods known to be employed by neurons, also exist in other cell types. We identify three major areas of long-distance communication: bioelectric signaling, tunneling nanotubes (TNTs), and macrophage modulation of networks, and draw comparisons about how these systems operate in the context of development and cancer. Bioelectric signaling occurs between cells through exchange of ions and tissue-level electric fields, leading to changes in biochemical gradients and molecular signaling pathways to control normal development and tumor growth and invasion in cancer. TNTs transport key morphogens and other cargo long distances, mediating electrical coupling, tissue patterning, and malignancy of cancer cells. Lastly macrophages maintain long distance signaling networks through trafficking of vesicles during development, providing communication relays and priming favorable microenvironments for cancer metastasis. By drawing comparisons between non-neural long distance signaling in the context of development and cancer we aim to encourage crosstalk between the two fields to cultivate new hypotheses and potential therapeutic strategies.

## Long-Distance Cellular Communication

Communication is essential at all levels of organism functioning, from coordinating morphogenesis during development to maintaining tissue homeostasis to prevent neoplasia. Sharing of information is an important method by which a population of cells can collect information from their environment and subsequently make individual and collective decisions about their behavior. Communication integrates highly competent individual cells toward a coherent multicellular anatomical outcome, allowing the myriad of cell types in the body to work in concert to maintain a complex organism. It is when this communication breaks down that disease states such as cancer arise. In metazoan organisms, neurons are possibly the most familiar example of implementation of long-distance communication networks. While these networks are classically associated with neurons, it is now known that many other cell types propagate and receive information at multiple size scales. It is important to understand the regulators of these non-neural communication networks, both to improve our understanding of the evolution of complex form and function, and to develop biomedical strategies for addressing various disease states.

Experimentalists strive to isolate biological phenomena into tractable systems to minimize noise and confounding off-target effects. This tendency has led to cell culture and tissue-specific promoter driven expression systems designed to target a cell, tissue, or process of interest. However, this approach requires the researcher to draw an arbitrary boundary of likely causal factors in space and time. We collect here evidence that factors outside of intuitive local spatial environments may play key underappreciated roles in both (1) early development, prior to the formation of the canonical avenues of long-distance communication, and (2) in cancer. In this review we avoid attempting to draw new hierarchical boundaries based on spatial distances. Instead, we use the term “long distance” not to imply a specific distance in micrometers but rather to generally refer to processes that are likely to fall outside of an experimentalist’s intuitive arbitrary spatial boundary.

### Long-Distance Communication During Early Development

Embryonic development is fundamentally a process of integration. Thousands of nearly interchangeable stem cells must adopt specific roles and find their correct location in the body. They must then form into tissues, which form into organs and organ systems and ultimately into a whole organism. And yet, establishing a bauplan is not the end of development; a growing organism must coordinate size increases between its disparate elements in unison while maintaining function and symmetry. Further, all organisms must maintain their shape *via* repair or regeneration in response to morphogenic challenges at all scales and employ long-distance communication to coordinate this large-scale morphogenic maintenance. Recent work in metamorphic tadpoles showing that bioelectric injury mirroring (BIM) signals encoding location and type of injury are transmitted from an amputated limb to its contralateral partner (Busse et al., 2018) provide a tantalizing glimpse of such communication and underscore the importance of examining seemingly local phenomena at the systems level.

Though most cell biology has focused on the local environment of a cell, each cell must integrate with many others, often at distances orders of magnitude greater than the immediate neighborhood of the cell, to generate a viable organism. Indeed, it is this anatomical spatio-temporal integration that separates a multicellular organism from a community of individuals. In stark contrast to an aggregate of single-celled organisms, which must simply optimize viability of its component individuals, metazoans must achieve a large-scale invariant anatomical goal generation after generation despite environmental variation. In adults, specialized organ systems, most notably the nervous, cardiovascular, and lymphatic systems, facilitate these interactions. Until recently, however, the mechanisms through which individual cells integrate over long distances, before these specialized communication channels have formed, have remained an open mystery for developmental biologists.

### Long-Distance Communication in Cancer

If development is a progression toward multicellularity and the formation of a specific organismal anatomy, cancer can be viewed as a breakdown of multicellularity and transformation of cells back into a unicellular state (Waddington, 1935; Needham, 1936; Moore et al., 2017). Cancer is a multifaceted and complex series of events including cell proliferation, migration, and colonization, each of which requires a high level of coordination to ensure “success” (i.e., formation of a tumor with the potential to form secondary tumors at distant sites). Although cancer is thought of as a collection of isolated cells pursuing single cell-level goals, it is increasingly clear that cancer cells do in fact communicate with each other and their surrounding environment, which may act over longer distances than previously realized. Considering the hypothesis that cancer can be viewed as a regression of somatic cells to a developmental or stem-like state (Ratajczak et al., 2018; Yang L. et al., 2020), it is useful to understand what long-distance communication cancer cells are capable of and how it may represent a pathological rebooting of multicellularity into tumors as a kind of novel organ-level system (Radisky et al., 2001; Egeblad et al., 2010; Thomas et al., 2016). Do cancer cells share information amongst themselves or with other cell types, and if so, how and at what distance? A better understanding of how cancer cells communicate with each other and their environment may inform new therapeutic strategies that seek to prevent, or reverse, the breakdown of the large-scale integration that keeps cell collectives working toward organogenesis and away from cancer and tumorigenesis (Levin, 2019).

### Goals of This Review

In this review we present an overview of recent findings uncovering a primitive non-neural network of connected cellular projections that enables cells to communicate at a distance. We discuss the potential of these networks to enable long-distance physiological signaling. Finally, we discuss evidence that macrophages control connectivity within this network. Throughout the review we draw on complementary examples from developmental biology and cancer literature, analyzing similarities between the two, and highlighting knowledge gaps in one field that may be filled with information from the other (Table 1). We thus hope to bring this emerging paradigm to the attention of a wide range of basic and applied researchers.

**TABLE 1:** Comparison of phenomena involved in local and long-distance communication in development and cancer.

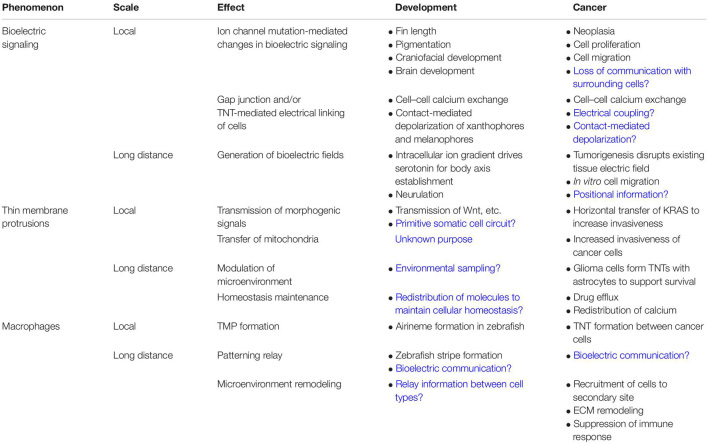

*Blue text indicates hypothesized roles of phenomenon.*

## Bioelectric Signaling

Bioelectric signaling refers to the control of cellular processes through the maintenance of ionic gradients across the plasma membrane, and the interpretation of the resulting voltage patterns by cells and their neighbors that regulates gene expression and cell behavior (Levin, 2021). Every cell of the body possesses an electric potential across their membrane, known as the membrane potential (*V*_*mem*_), and can generate and receive bioelectric signals. The membrane potential is regulated through the expression and activity of ion channels/transporters, ionic composition of the extracellular milieu, and the presence of tissue-level bioelectric gradients (Wright, 2004). Bioelectric signaling is an important controller of many essential processes at the single cell, multi-cell, and tissue levels (Kirson et al., 2007; Funk, 2015). Importantly, it is becoming increasingly clear that in both neural and non-neural cells bioelectric is not merely a physiological homeostat, but plays instructive roles in cell proliferation, differentiation, and apoptosis by establishing biochemical gradients, altering gene expression, and modulating cell signaling (Pai et al., 2016; Ricci and Srivastava, 2018). While signaling *via* changes in *V*_*mem*_ takes place through cell:cell electrical synapses known as gap junctions (Palacios-Prado and Bukauskas, 2009; Mathews and Levin, 2017), it is not merely a local, cell-level phenomenon; long-distance bioelectric signaling has been observed in bacterial biofilms to facilitate communication within (Prindle et al., 2015) and between (Humphries et al., 2017) species, as well as to enable memory (Yang C. Y. et al., 2020), underscoring its universality as a biological information processing paradigm.

### Neurons as an Example of Long-Distance Bioelectric Signaling

Neuronal signaling is perhaps the best-studied example of long-distance biophysical signaling. Neurons control their membrane potential *via* ion pumps and channels, transmit these signals over very long distances *via* highly specialized axonal projections, and interface with receiving cells *via* gap junctions or targeted secretion. With a growing number of examples of thin membrane protrusions (TMPs) (Yamashita et al., 2018) it is becoming increasingly clear that non-neural cells possess primitive versions of each of these components which will be discussed in the following sections. Further, mutations in any of these components can have profound developmental and disease effects indicating that these long-distance non-neural bioelectric signals are instrumental to normal development and somatic cell functioning (Bates, 2015).

### Development and Bioelectric Signaling

Endogenous electric fields have been identified during even very early normal development (Funk, 2015). An intracellular ion gradient established by uneven distribution of ion channels and pumps at the first few cell cleavages drives electrophoresis of serotonin through gap junctions in the early vertebrate embryo, thereby establishing the left-right axis (Levin and Mercola, 1998; Levin et al., 2002; Adams et al., 2006). During neurulation in salamanders, medio-lateral trans-epithelial potential gradients have been measured around the closing neural folds (Shi and Borgens, 1995). Though no function has been directly attributed to these fields, galvanotactic behavior has been observed in neural crest cells (Nuccitelli and Erickson, 1983), which migrate along this axis. These examples implicate endogenous electric fields as a general long-distance organizational paradigm, and better understanding them will both increase our understanding of large-scale organization and how breakdown of these processes can drive tumor progression.

Bioelectric signaling fundamentally differs from growth factor-based signaling in that information is encoded not by specific proteins but by physiological parameters established by the action of many diverse ion channels. The functional importance of these signals is demonstrated by genetic work in zebrafish and *Xenopus* which has implicated ion channels in long-distance communication, with mutations in ion channels controlling fin length (Perathoner et al., 2014; Daane et al., 2018), pigmentation (Blackiston et al., 2011; Inaba et al., 2012), craniofacial development (Vandenberg et al., 2012; Adams et al., 2016), cell migration (McMillen et al., 2020), and brain development (Pai et al., 2015b, 2018, 2020) over large scales. Endogenous bioelectric prepatterns are essential for normal morphogenesis. In frogs, depolarization of a small subset of glycine-channel expressing cells leads to melanocyte hyperproliferation and hyperpigmentation of the entire tadpole (Blackiston et al., 2011, 2017). Conversely, in zebrafish a mutation in the inward rectifying potassium channel gene *KCNJ13* encoding Kir7.1 renders melanocytes incompetent to receive bioelectric signals from xanthophores and dramatically alters the pigmentation pattern of the mutant animal (Iwashita et al., 2006; Inaba et al., 2012). Similarly, mutation of potassium channel genes induces dramatic fin overgrowth in zebrafish leading to striking “long-finned” animals (Perathoner et al., 2014; Daane et al., 2018). Further, mutations in ion channels have been attributed to developmental abnormalities in such diverse systems as fly wings (George et al., 2019) and craniofacial development in frogs (Vandenberg et al., 2011; Adams et al., 2016; McMillen et al., 2020) and mice (Belus et al., 2018). These examples underscore the magnitude of developmental consequences that can arise from perturbation of long-distance signaling pathways such as bioelectricity.

Information generated by the activity of ion channels must be transmitted to other cells in order to act over long distances. Neurons accomplish this *via* highly specialized synapses that enable tightly controlled transmission of information between cells either by targeted vesicular release or by gap junctions. Gap junctions play a similar primitive function in non-neural cells. Differentially charged cells connected by gap junctions can establish stable electric fields, which in turn can drive inter-cellular electrophoresis of charged chemical messengers like serotonin or calcium (Levin et al., 2002; Fukumoto et al., 2005). Many gap junctions are also voltage-gated, enabling direct control of information transfer by alteration of membrane potential (Mathews and Levin, 2017) – a feedback property that allows sophisticated computation to occur in gap junction-coupled circuits (Cervera et al., 2017, 2018, 2019; Pietak and Levin, 2017). Importantly, such electrophoretic control of signal movement may not be restricted to adjacent cells, as a subset of TMPs termed tunneling nanotubes (TNTs) directly linking the cytoplasm of connected cells shows the potential to electrically link distant cells (Wang et al., 2010; McKinney et al., 2011; Wang and Gerdes, 2012). Further, the prevalence of voltage gated calcium channels [which have been identified as early as the two-cell stage in zebrafish (Sanhueza et al., 2009)] in non-neural cells suggests that transmitted membrane potential changes may be sufficient to induce calcium-dependent morphogenetic events without the need for electrophoresis. Such channels have been implicated in potassium channel-dependent increase in cell migration, which may cause long-distant effects when these migratory cells move to ectopic locations (McMillen et al., 2020). Another identified bioelectric mechanism is contact-mediated depolarization, which has been described between xanthophores and melanophores in developing zebrafish skin (Inaba et al., 2012; Irion et al., 2014; Yamanaka and Kondo, 2014). Moreover, there remain several examples of long-range bioelectric phenomena, such as the long-distance normalization of tumors (Chernet and Levin, 2014), bioelectric repair of genetic teratogenesis (Pai et al., 2020), and BIM (Busse et al., 2018), whose mechanisms remain to be uncovered.

### Cancer and Bioelectric Signaling

Cancer bioelectricity is a fledgling field that may take inspiration from some of the phenomena observed in development (Chernet and Levin, 2013). It has been known for decades that gap junctional communication is as important for carcinogenesis (Mesnil et al., 2005; Trosko, 2005, 2007; Leithe et al., 2006; Kandouz and Batist, 2010; Defamie et al., 2014) as it is for coordinating cell behavior in normal development. There is increasing evidence that ion channel-mediated effects in cancer are important for neoplasia and tumor progression. Cancers of many different types over or under express a substantial number of ion channels and have an altered resting membrane potential (Roger et al., 2003; Diaz et al., 2007; Djamgoz et al., 2019; Wang et al., 2019). Drug- or ion channel-induced depolarization of instructor cells in *Xenopus* embryos not subjected to carcinogens, DNA damage, or oncogene induction leads to a metastatic-like phenotype, including massive over-proliferation and invasion of blood vessels and brain by genetically normal melanocytes (Blackiston et al., 2011; Lobikin et al., 2015). Importantly, the effect is not cell-autonomous and takes place at long distances: depolarization of instructor cells in the head is sufficient to transform melanocytes in the tail (Lobikin et al., 2012), and forced hyperpolarization of cells on one side of the body is sufficient to prevent conversion of melanocytes on the opposite side (Blackiston et al., 2011). Ion channel and gap junction mutations also have consequences in cancer cell proliferation and migration; blocking channels such as voltage-gated sodium or potassium can reduce cancer cell malignancy *in vitro* and in animal models (Roger et al., 2003; Wang et al., 2019). The concept that a mutation in an ion channel, such as Kir7.1 in zebrafish melanocytes, can block bioelectric signaling has interesting implications for cancer (Blackiston et al., 2011; Lobikin et al., 2015; Lobo et al., 2017). That is, it raises the possibility that changing the bioelectric phenotype of cancer cells could block their ability to receive regulatory bioelectric signals from the surrounding tissue, electrically isolating the cancer cell. It has been hypothesized that this loss of bioelectric communication between cancer cells and the surrounding normal tissue may help define the boundary between self and non-self in cancer cells (Levin, 2019).

Although cancer is generally associated with a loss of gap junctions (Loewenstein and Kanno, 1966; Yamasaki et al., 1987; Trosko and Ruch, 1998; Blackiston et al., 2011; Aasen et al., 2017), there is evidence that, like during zebrafish development, cancer cells do exchange ions such as calcium (Ariazi et al., 2017; Valdebenito et al., 2018). This suggests that cancer cells may use bioelectric signaling to function in a similar fashion as instructor cells in the embryo. There is evidence that cancer cells can form connexin-containing gap junctions using TNTs, mediating calcium flux between cancer cells (Ariazi et al., 2017; Valdebenito et al., 2018), which has implications for the control of many downstream pathways involving cell proliferation and migration (Osswald et al., 2015; Lock et al., 2016; Valdebenito et al., 2018). Cells connected by TNTs may also be electrically coupled, but this an understudied area in cancer (Wang et al., 2010; Wang and Gerdes, 2012). In glioma it has been shown that TNTs can propagate waves of calcium which may promote glioma proliferation *in vivo* (Osswald et al., 2015), but it is unclear if this occurs in cancers of a non-neural origin. Cancer cells are also typically more depolarized than their normal counterparts, raising the possibility that they use this feature to induce depolarization in neighboring cells in a process akin to the contact-mediated depolarization during development. Since cancer cells can transmit a variety of signals and molecules to other cells, it is conceivable that they may also do so with bioelectric signals.

At the tissue level, the electric field generated by tumors themselves have a different potential than surrounding normal tissue which is detectable at the surface of the tumor (Burr, 1941; Wu et al., 2013; Nakajima et al., 2015). There is also evidence that extracellular ion concentrations are altered in tumors and pre-cancerous cysts, particularly sodium and potassium (Sişman et al., 2009; Djamgoz et al., 2019) but it is unknown whether this is a significant factor in bioelectric-mediated cancer cell behavior. In epithelial tissues, the formation of a tumor disrupts the normal transepithelial potential in a process akin to epithelial wounding (Mycielska and Djamgoz, 2004; Ren et al., 2019). This disruption may cause a loss of communication between the tumor and surrounding tissue, stimulating tumor progression and metastasis. In tadpoles, metastatic transformation of normal melanocytes can be induced by depolarizing a specific cell population distant from the melanocytes (Lobikin et al., 2012), and conversely, human oncogenes can be prevented from inducing tumors in *Xenopus* by forced hyperpolarization (Chernet and Levin, 2014; Chernet et al., 2016). An especially exciting observation is that induction of hyperpolarization in only a small population of instructor cells located far from the oncogene-containing cells was able to revert the cancer phenotype (Chernet and Levin, 2014). Furthermore, electrotaxis, the migration of cells in response to an electric field, has been reported in many cancer cell types *in vitro* and is hypothesized to promote tumor cell migration (Pu et al., 2007; Wu et al., 2013; Nakajima et al., 2015). It is currently unknown how long range these signals can be *in situ*, or which cell type can be affected. One intriguing possibility is that the differing bioelectric signature helps to establish the boundary between self and non-self in cancer cells, driving them toward a more tumorigenic state. Future work will need to explore if abnormal electric fields generated from tumors can directly transmit bioelectric signals or induce the production of other signaling types to long-distance areas of the organism.

## Thin Membrane Protrusions

### Phenomenology of Thin Membrane Protrusions

The discovery of TMPs in non-neural cells greatly extends our perception of the distance over which cells can send and receive cell-derived signals. It has been proposed that TMPs are analogous to neuronal axons due to their ability to transport glutamate and calcium over long distances (Ramírez-Weber and Kornberg, 1999; Nussenzveig, 2019; Cordero Cervantes and Zurzolo, 2021). Several types of long-distance projections have been identified with unique structural properties (Yamashita et al., 2018). Projections that directly link two cells without an intervening membrane boundary are called TNTs, while projections with closed ends that do not directly connect the cytoplasm of the two cells are called cytonemes. Interestingly, recent work has implicated membrane depolarization and glutamatergic signaling at cytoneme synapses (Huang et al., 2019) providing further analogy between neural and non-neural long distance signaling. A third type of projection has recently been discovered consisting of membrane-enclosed vesicles tethered to sending cells *via* projections and named airinemes (Eom et al., 2015). While the structural makeup of these types of TMPs vary, each functions as a channel by which long-distance information can directly be transmitted between cells. The study of TMPs provides an interesting variation on the theme of signaling at two scales: the direct nature of TMP connection facilitates of local cell–cell interactions, while the long distance over which they span enables macroscale coordination.

### Thin Membrane Protrusions in Development

The presence of TMPs has been reported in mammalian cells, plants (aka plasmodesmata), and *Drosophila* (aka cytonemes), suggesting that they are a highly conserved feature for intercellular communication. TMPs have been identified as early as the 32-cell stage in frog embryos (Danilchik et al., 2013), long before differentiation of the first neurons, and they have also been extensively described in the migrating neural crest (Teddy and Kulesa, 2004; McKinney et al., 2011). These early manifestations implicate TMPs in the first events of embryonic development including axis formation, germ layer induction, neural specification, and neural crest migration. Though there is limited functional data on these structures during development (Luz et al., 2014; Stanganello et al., 2015), elegant work in *Drosophila* has shown that cytonemes can transduce many key morphogenic signals involved in these early developmental processes including Wnt (Huang and Kornberg, 2015), FGF (Ramírez-Weber and Kornberg, 1999), BMP (Roy et al., 2014), and even the canonically short range Hedgehog (Rojas-Ríos et al., 2012) and Notch pathways (Huang and Kornberg, 2015). Interestingly, it has further been suggested that a single cell can produce cytonemes for specific signaling pathways (Roy et al., 2011) indicating that cells may play an active role in sampling their environment. These findings underscore the importance of looking beyond the intuitive local spatial boundaries of a cell to understand the activity of well-studied pathways. Indeed, the diversity, complexity and distance of these connections provides a tantalizing suggestion that somatic cells may compose primitive regulatory circuits reminiscent of those found in neurons.

Better understanding of TMPs has the potential to transform our understanding of cells, allowing us to see them as components of systems as well as individual functional units. TMPs provide a compelling putative mechanism for the unexpectedly long-range effects of physiological signaling. Alteration of ion channels (Chernet and Levin, 2014; Pai et al., 2015a, 2020) or gap junctions (Chernet et al., 2014) induces physiological responses at sites distant from the manipulation, and amputation has been shown to induce changes in potentiometric dye localization on the contralateral limb of pre-metamorphic tadpoles (Busse et al., 2018). Work in cultured neural crest cells as well as in glioma (Kirson et al., 2007) has shown that distant cells connected by TNTs can be electrically coupled (Wang et al., 2010; Wang and Gerdes, 2012) *via* gap junctions, providing a possible mechanism for these long-distance effects, though this model lacks *in vivo* testing.

### Thin Membrane Protrusions in Cancer

In addition to their role in normal development, TMPs have been implicated in disease progression. TMPs fundamentally increase the potential for cells to control others beyond their local environment. However, this increased range of action can also be leveraged by neoplastic cells to wreak havoc throughout the body. TNTs facilitate communication between two cancer cells or cancer cells and their surrounding stroma to both propagate the cancer phenotype and support survival and growth of a tumor. The formation of cancer cell TNTs, thought to be in response to stimuli such as growth factors and hypoxia (Prindle et al., 2015; Mathews and Levin, 2017), has been demonstrated in many cancer cell lines *in vitro* and there is mounting evidence that they are also present *in vivo* (Osswald et al., 2016; Weil et al., 2017; Desir et al., 2019). TNT formation has been linked to a stress response thought to be an attempt to reestablish homeostasis; for example, following brain injury, astrocytes will form TNTs with neurons in order to provide them with pro-survival signals (Osswald et al., 2015). Cancer cells appear to coopt this stress-induced TNT formation as a method to communicate with neighboring cells, shaping their surrounding environment to support tumor growth and progression. Understanding the function of TNT based tumor networks may provide fresh insights for cancer therapies targeting TNT disruption.

There is evidence that cancer cell TNTs may both directly fuse with the recipient cell, or form connexin-containing gap junctions (Ariazi et al., 2017; Valdebenito et al., 2018). The ability of TNTs to transport a wide variety of cargo between cells (mitochondria microRNAs, cytokines, growth factors, calcium, and even chemotherapeutic molecules can be transported *via* TNTs) is advantageous to cancer cells in several ways. Those that form with gap junctions have been shown to mediate calcium flux between cancer cells, which has implications for the control of many downstream pathways involving cell proliferation and migration (Osswald et al., 2015; Lock et al., 2016; Valdebenito et al., 2018). The best characterized example of this is in glioma and particularly work done in the Winkler lab. Glioma cells form TNTs with nearby astrocytes which support glioma survival (Osswald et al., 2015). Furthermore, glioma TNTs were reported to contain a structure at the tip that resembles the growth cone of neurons, which is hypothesized to aid the glioma cell invasion in the brain (Osswald et al., 2015). Cancer cells also use TNTs to increase their invasiveness *via* cytoplasmic transfer of different cargo. The *in vitro* transfer of mitochondria between bladder cancer and normal bladder cells results in increased invasiveness of bladder cancer cells in culture and *in vivo* (Lu et al., 2017). In addition, the horizontal transfer of KRAS between colorectal cancer cells was shown to both introduce intra-tumoral heterogeneity and increase invasiveness (Desir et al., 2019). Cancer cell TNTs have also been observed to facilitate redistribution of chemotherapeutic drugs between cells *in vitro* in a buffering-like effect. In pancreatic and ovarian cancer, dosing cells with doxorubicin increased the formation of TNT and resulted in drug efflux (Desir et al., 2018). A similar phenomenon has been observed in the brain: networks of glioma cells connected by TNTs are more resistant to radiotherapy and chemotherapy (Weil et al., 2017).

Aside from local cell–cell transfer, available evidence suggests that cancer cells may even use TNTs to exchange information with distant cells, forming communication networks on a macroscopic level not unlike neural networks (Kirson et al., 2007; Palacios-Prado and Bukauskas, 2009). Interestingly, cancer cells seem to have co-opted the use of TNTs to redistribute molecules between cells to maintain homeostasis as a means of survival. When neurons are injured, nearby astrocytes form TNTs which exchange calcium and mitochondria with the injured neurons to support their survival (Osswald et al., 2016; English et al., 2020). In culture, stressed rat hippocampal astrocytes form TNTs which develop toward unstressed astrocytes in a p53-dependent process (Wang et al., 2011). In a process reminiscent of this, reactive astrocytes have been observed to form TNTs with melanoma cells following chemotherapy which transfer calcium from the melanoma cells, reducing the effect of chemotherapy-induced calcium-dependent apoptosis (Lin et al., 2010; Osswald et al., 2015). This ability of cancer cells to use TNTs to form large multicellular networks is a striking example of collective communication which enables the coordinated survival of cancer cells. Targeting TNT formation between cancer cells and stroma may disrupt the network communication and help to eliminate drug resistance developed by some cancer cells.

### Implications of Cancer Thin Membrane Protrusions for Developmental Biology

Though our understanding of the *in vivo* developmental functions of TMP-mediated communication remains limited, it will be interesting to see how the long-distance transforming capacity of TMPs observed in cancer cells manifests during development. Of particular interest is the horizontal transfer phenomenon observed with KRAS (Desir et al., 2019). Photoconversion tracing studies (McKinney et al., 2011) have shown that an exogenous protein (KikGR) can be trafficked between neural crest cells, indicating that long-distance protein transmission is not limited to KRAS or to cancerous cells. This phenomenon could have profound implications for gene expression analyses. Thorough spatial gene expression atlases have been established in several model organisms [Xenbase (Karimi et al., 2018), Zfin (Ruzicka et al., 2019), the mouse Gene Expression Database (GXD) (Smith et al., 2019), and flybase (Larkin et al., 2020)], but protein localization is far more sparsely mapped due to the relative difficulty of producing specific antibodies. If, however, proteins are readily trafficked to distant cells from the site of their transcription then these atlases may provide an incomplete picture, underscoring the importance of validating gene expression analyses with immunohistochemistry when possible. Further, the spatial decoupling of mRNA expression and protein localization could have serious implications for the growing field of single-cell analysis, as two cells may have indistinguishable transcriptomes but very different proteomes if they receive proteins from distant sites.

## Macrophages

In adult organisms, long-distance communication is mediated by specialized organ systems, notably the neural, circulatory, and lymphatic systems. Likewise, in embryos there is a growing body of evidence that specialized cells transmit information and establish connections between distant cells. TMPs and bioelectric signaling give cells the means to establish long-distance communication; but without regulation of these connections, network formation becomes problematic. In the brain, synaptic connections are precisely pruned by the activity of microglia (tissue resident macrophages), and this pruning of excess connections is essential to normal brain function (Paolicelli et al., 2011). In non-neural systems there is evidence that TMP-mediated connectivity, bioelectric or otherwise, may likewise be regulated by macrophages. Moreover, as with the phenomena discussed in the previous two sections, cancer cells can leverage the connectivity modulating power of macrophages to affect changes at distant sites.

### Macrophages in Development

**FIGURE 1:**
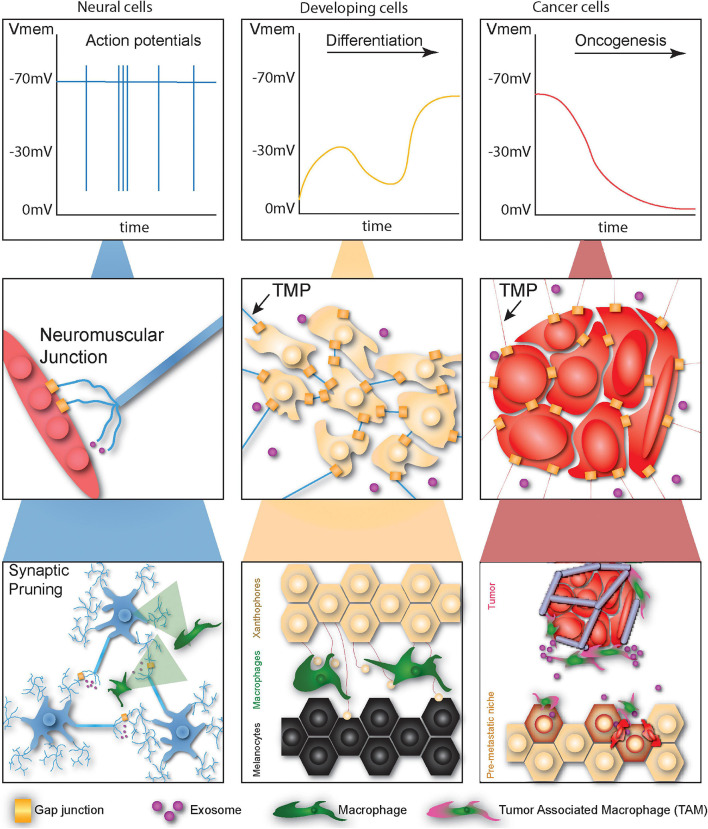
Cells undergoing developmental or tumorigenic programs employ primitive non-neural communication methods for signaling at different scales. At the physiological scale neural cells signal *via* fast high-magnitude changes in membrane potential. Developing cells communicate *via* membrane potential fluctuations, though these changes are far slower and lower in magnitude. Cancer cells tend to depolarize over time, facilitating oncogenesis. At the local scale, neurons communicate directly with myocytes *via* gap junctions or translate bioelectric signals into chemical signals *via* vesicular release. Normal and cancerous non-neural cells likewise communicate at long distances *via* thin membrane protrusions (TMPs), gap junctions, and exosomes. At long-distance neurons form networks capable of complex computation regulated by the pruning activity of macrophages. Macrophages also establish networks of signaling projections between different types of skin cells during development and facilitate priming of the metastatic niche during cancer metastasis.

Macrophages have long been known to play key roles during normal development (Wynn et al., 2013) and regeneration (Godwin et al., 2013). Exciting recent work with post-embryonic zebrafish skin has implicated macrophages in patterning of the stripes from which zebrafish derive their name in what has been labeled a “macrophage relay” (Eom and Parichy, 2017; Guilliams, 2017). Macrophages facilitate communication between two distinct pigment cells: black melanophores and yellow xanthophores. They extract vesicles from xanthophores which they then traffic, intact, to melanophores while leaving behind airinemes. When this process is disrupted by depleting macrophages, melanocytes readily invade the xanthophore domain, leading to improper development of the normal stripe pattern (see Figure 1).

Interestingly, another mechanism of long-distance communication described in this review, bioelectric signaling, may also be involved in the macrophage relays. Ion channels and inter-cellular bioelectric communication have been implicated in zebrafish stripe formation, though macrophages have not been linked to these processes (Iwashita et al., 2006; Inaba et al., 2012). As this work was completed prior to the identification of the macrophage relay, however, it may well be that such a connection remains to be uncovered. Indeed, there is also a growing body of evidence that bioelectric manipulation either *via* chemicals (Paré et al., 2017) or removal of central nervous system (Herrera-Rincon et al., 2020) input can alter macrophage behavior, and it is worth investigating whether this phenomenon may be leveraged as a tool for controlling metastasis.

### Macrophages in Cancer

Cancer cells communicate with many cell types in their surrounding parenchyma as a means of promoting tumor growth and metastasis, including endothelial cells, fibroblasts, adipocytes, neurites, and immune cells. The crosstalk that occurs between cancer and immune cells, particularly macrophages, is a striking example of long-distance communication between two different cell types. Broadly, this crosstalk is involved in remodeling the microenvironment toward a pro-tumorigenic state and the recruitment of cells to distant sites in the body (i.e., recruitment of macrophages to the tumor, or tumor cells to a site of metastasis). Understanding these mechanisms in the context of cancer may lead to insight into the long-distance crosstalk in other systems such as embryogenesis.

It has long been known that chronic infiltration of a tissue by macrophages and inflammation can promote neoplastic transformation, although the exact mechanism remains unclear. Early on in tumor formation, tumor cells secrete factors, cytokines, and ECM components into their environment in response to stimuli such as hypoxia to attract macrophages, causing their activation and polarization (Kim et al., 2009; Park et al., 2019). Tumor-associated macrophages (TAMs) are converted to the M2 phenotype (anti-inflammatory, proangiogenic) through tumor cell cytokine secretion and activation of the Wnt pathway (Ojalvo et al., 1950). Cancer cells also recruit macrophages to the tumor by secreting exosomes containing IL-8, VEGF, HGF, and CD44 mRNA that re-educate nearby monocytes, which differentiate into TAMs once they enter the tumor stroma (Baj-Krzyworzeka et al., 2006; Richards et al., 2013). It has been suggested that TNT formation may be linked to tumor–macrophage interactions (Hanna et al., 2019); TNT formation dependent on macrophage-secreted M-sec and EGF has been shown to increase 3D breast cancer invasion *in vitro* (Carter et al., 2019). Once TAMs are recruited to the tumor site, they in turn secrete factors that act on tumor cells to support their growth and metastasis (Coussens et al., 2000) and suppress the immune response against cancer cells (Solinas et al., 1950; Pathria et al., 2019). There is also evidence that TAMs can reduce the chemosensitivity of cancer cells, with one study demonstrating that miRNA contained in exosomes secreted by M2 macrophages conferred cisplatin resistance to gastric cancer cells *in vivo* (Zheng et al., 2017). Interestingly, TAMs also promote pro-metastatic changes in the tumor microenvironment in response to chemotherapy treatment (Sanchez et al., 2019).

One of the more striking examples of long-distance communication between TAMs and cancer cells is the priming of the pre-metastatic niche. For cancer cells to disseminate from the primary tumor and colonize a secondary location, they must be able to exit the circulation, migrate, and survive in the new site. Cancer cells can induce the early priming of these secondary sites, forming what is known as the premetastatic niche, before leaving the primary tumor. For example, pancreatic ductal carcinoma cells secrete exosomes from the primary tumor site which circulate to the liver where they fuse with Kupffer cells (macrophages of the liver) and cause activation of fibrotic and pro-inflammatory pathways that support metastases (Costa-Silva et al., 2015). TAMs also participate in priming of the premetastatic niche, secreting inflammatory cytokines which increase the success of lung metastasis (Nguyen et al., 2009). These observations suggest that cancer cells can play an instructor cell-like role, signaling to stromal cells to model microenvironments into favorable conditions in advance of the cancer cell arrival. Moreover, TAMs, aside from secreting factors that enter the circulation, are also directly recruited to the premetastatic niche and their presence is necessary for cancer cell survival at the secondary metastatic site (Hiratsuka et al., 2008; Gil-Bernabé et al., 2012; Kim et al., 2019).

### Implications of Long-Distance Macrophage Communication

Better understanding of how macrophages mediate long-distance communication during development may provide key insights for deciphering their role in cancer. The observation that breakdown in this process leads to invasion of melanocytes in zebrafish provides tantalizing evidence that this process may function in metastasis of highly aggressive cancers such as melanoma. Just as TAMs mediate communication between tumors and pre-metastatic niches, during stripe formation macrophages mediate communication between two distant and distinct cell types. The mechanisms by which macrophages identify and distinguish these two cell types may point to more general mechanisms by which TAMs preferentially target certain cell and tissue types. Similarly, the importance of airinemes and other projections (Eom et al., 2015) in this process may lead to better understanding of the pathological roles of these structures in metastasis.

## Concluding Remarks

It is important to understand methods of long-distance communication in non-neural networks. Herein we discussed recent insights into non-neural networks for communication at multiple scales and compare the two contexts of development and neoplasia to suggest what each field may learn from the other, summarized in Figure 1 and Table 1. Bioelectric signals can electrically couple cells during development, providing high level instructions for patterning and morphogenesis, and in cancer initiate signaling cascades to increase malignancy. TNTs offer the ability to control cell behavior beyond the local environment, sharing bioelectric, and morphogenic signals between cells during development and a variety of cargo in cancer cells to control cellular identity and shape the microenvironment. Lastly, we discussed the ability of macrophages to regulate long distance networks between cells in development and cancer, acting as relay cells to facilitate crosstalk between different cell types during development and establishing metastatic niches for cancer cells in distant organs. Although in most of the examples we provided these non-neural networks participate in different functions in development versus cancer, comparing the mechanisms of these networks is a valuable tool for both fields. Clearly development is a coordinated effort among cells to achieve large-scale patterning and tissue formation, but cancer, while not a coordinated effort of the body, can be considered coordinated within the cancer “organism”; there is simultaneously a breakdown of normal signaling but also the establishment of new communication routes with the recruitment of macrophages and formation of TMPs. Understanding how communication in these non-neural networks occurs will allow us to control it in disease states of both embryonic and post-natal organisms.

The long-distance phenomena described here may have profound implications for many different fields of biology. Developmental biologists would be well advised to look for novel players in well-studied processes at much greater distances than previously examined, as underscored by the recent discover of bioelectric injury mirroring (Busse et al., 2018). Disease researchers may find pathological sources in surprising accessory cell types located far from the affected cells. Clinically, the long-distance phenomena we review here may enable diagnosis or treatment of inoperable disease at readily manipulable surrogate sites. And, finally, the prevalence of long-distance communication may force us to re-evaluate our notions of cell autonomy as we uncover more evidence that a cell’s behavior is not a local phenomenon, but rather an emergent integration of information from throughout the body.

## Author Contributions

PM and SP conceived of the manuscript topic and drafted the manuscript. MO and ML critically revised it. All authors contributed to the article and approved the submitted version.

## Conflict of Interest

The authors declare that the research was conducted in the absence of any commercial or financial relationships that could be construed as a potential conflict of interest. The reviewer MZ declared a shared consortium with one of the author ML to the handling editor.

## Publisher’s Note

All claims expressed in this article are solely those of the authors and do not necessarily represent those of their affiliated organizations, or those of the publisher, the editors and the reviewers. Any product that may be evaluated in this article, or claim that may be made by its manufacturer, is not guaranteed or endorsed by the publisher.
